# Pipit: visualizing functional impacts of structural variations

**DOI:** 10.1093/bioinformatics/btt367

**Published:** 2013-06-25

**Authors:** Ryo Sakai, Matthieu Moisse, Joke Reumers, Jan Aerts

**Affiliations:** ^1^Department of Electrical Engineering-ESAT, KU Leuven, SCD-SISTA, Leuven 3001, Belgium, ^2^Future Health Department, iMinds, Leuven 3001, Belgium, ^3^Vesalius Research Center, VIB and KU Leuven, Leuven 3000, Belgium, ^4^Laboratory of Translational Genetics, Department of Oncology, KU Leuven, Leuven 3000, Belgium and ^5^Janssen Infectious Diseases–Diagnostics, Janssen Pharmaceuticals, Beerse 2340, Belgium

## Abstract

**Summary:** Pipit is a gene-centric interactive visualization tool designed to study structural genomic variations. Through focusing on individual genes as the functional unit, researchers are able to study and generate hypotheses on the biological impact of different structural variations, for instance, the deletion of dosage-sensitive genes or the formation of fusion genes. Pipit is a cross-platform Java application that visualizes structural variation data from Genome Variation Format files.

**Availability:** Executables, source code, sample data, documentation and screencast are available at https://bitbucket.org/biovizleuven/pipit.

**Contact:** ryo.sakai@esat.kuleuven.be

**Supplementary information:** Supplementary data are available at *Bioinformatics* online.

## 1 INTRODUCTION

Structural variation is defined as a change of genomic DNA greater than 1 kb in size and can be either balanced or unbalanced (Stankiewicz and Lupski, 2010). A structural variation may be benign, it may influence phenotypes, it may predispose to or cause diseases, and it may be transmitted to next generations. In addition, it may also result in the formation of new transcripts through gene fusion or exon skipping when breakpoints disrupt gene structures (Feuk *et al.*, 2006). Understanding the structural change in the genome, as well as its functional impact, is critical for studying phenotypic variations and genetic diseases in human and model organisms (Korbel *et al.*, 2007).

When structural variations are studied, the structure of these variants are usually visualized by encoding breakpoints on a linear or circular layout (Herbig *et al.*, 2012; Krzywinski *et al.*, 2009). Other visual encodings such as dot plot and graph representations show the changes in the reference genome introduced by the structural rearrangement (Nielsen and Wong, 2012). These visual encodings pose two challenges in analysis of structural variation data. First, they focus on size and position, rather than the consequence of structural rearrangement. Second, they introduce an implicit correlation between size and effect, whereas small structural variations may have more severe effects than some large variations. Therefore, an effective visual encoding of structural and copy-number variations based on functional units is essential to gain insights into impact on human health and disease.

We introduce an exploratory visualization tool, named Pipit, which uses a novel gene-centric visual encoding to examine how gene structures individually are affected by structural variants. Pipit takes a Genome Variation Format file as input and encodes an affected gene structure as a disk, showing how it is modified and oriented towards other genes. By means of this data abstraction, the focus of the structural variants is shifted from the genomic distance to the biologically relevant feature, i.e. the underlying genes. This encoding enables swift visual inspection of genes that are affected by structural variants, which may be examined further if deemed interesting. Not constrained or biased by variation sizes, Pipit permits in-depth analysis and exploration of structural variation data.

## 2 FEATURES

Pipit is an interactive visualization tool developed in Processing, an open source programming language and integrated development environment based on Java. The executables are available for Linux, Mac OS X and Windows. The input file is in Genome Variation Format because it is a well-standardized format for genomic structural variation data, extended from the Generic Feature Format (Reese *et al.*, 2010), and both the European Bioinformatics Institute and the National Center for Biotechnology Information (NCBI) curate, archive and make data publicly available in this format via DGVa (http://www.ebi.ac.uk/dgva) and dbVar (http://www.ncbi.nlm.nih.gov/dbvar), respectively (Lappalainen *et al.*, 2013).

**Fig. 1.**
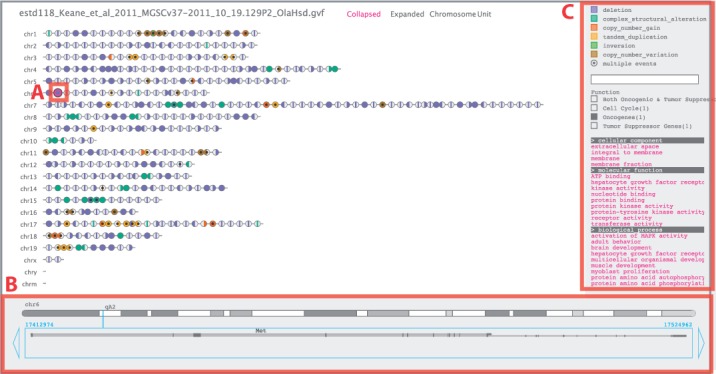
Pipit visualizing the structural variation data of the mouse genome (estd118). (**A**) The deletion of the *Met* gene on chromosome 6 is selected. (**B**) The associated genomic information of the region is shown on the bottom panel. (**C**) Categories of the known oncogenes for the mouse are listed, and the *Oncogenes* category is selected. GO terms associated with affected genes are listed below on the right panel

Pipit also uses the gene track, cytoband and gene ontology (GO) information obtained from the UCSC table browser database (Karolchik *et al.*, 2004). The current version supports the data from human (NCBI build 36 and 37) and mouse (NCBI build 37/mm9 and GRCm build 38/mm10), but the user can load the data for other model organisms. In addition, the user can load a comma-separated values file containing the Ensembl gene ID and ordinal or categorical information, such as haploinsufficiency scores (Huang *et al.*, 2010) and known oncogenes (Fig. 1 and Supplementary Material).

Each affected gene is represented as a disk and filled according to which part of its structure is influenced by a structural variation (Fig. 1; see Supplementary Material). Structural variant types are based on the data file and colour coded as shown on the right panel. Unaffected genes are compressed into a line connecting affected genes. The default promoter length upstream of the gene sequence can be set when loading the data.

There are four layouts to explore the structural variation data. The default view is the *collapsed ordered gene view* (Fig. 1). In this view, a coloured disk may represent an affected gene or consecutively ordered genes that are affected by the same type of structural variation. In the *expanded view*, all affected genes are individually visualized. The *chromosome position view* shows affected variants mapped to their genomic positions. Lastly, the *unit plot view* visualizes affected genes by their type of structural variant event, such as deletion, tandem-duplication and so forth (see Supplementary Material).

When a disk unit is selected (Fig. 1A), the underlying genes and structural variation events are shown on the bottom panel, along with the chromosome with cytobands and transcripts with their exonic regions coloured in dark grey (Fig. 1B). The gene name shown in this panel links to the Ensembl browser and displays the genomic region. In the right panel (Fig. 1C), the coloured square boxes for each structural variant types serve as radio buttons to hide or show a selected type of variant. The text field below searches for a specific gene amongst affected genes. GO terms associated with affected genes are listed, and conversely selecting a GO term highlights associated genes in the main view. A screenshot can be saved as a PDF by pressing the ‘p’ on the keyboard.

## 3 DISCUSSION

Pipit introduces a novel visualization paradigm and user interaction method to examine structural variants based on the affected gene region. It facilitates the study of structural variants from a gene-centric perspective to investigate various events, for instance, how known dosage-sensitive genes are affected or whether gene fusions are formed. Future work includes extending the functional unit to encompass important regulatory elements as elaborated in the ENCODE project (Birney *et al.*, 2007). Additionally, functions to compare multiple samples from various data formats, such as Variant Call Format (Danecek *et al.*, 2011), and options to incorporate other conventional linear or circular representations are essential for more comprehensive study of structural variants.

*Funding*: iMinds [SBO 2012], University of Leuven Research Council [SymBioSys PFV/10/016, GOA/10/009] and European Union Framework Programme 7 [HEALTH-F2-2008-223040 ‘CHeartED’].

*Conflict of Interest:* none declared.

## Supplementary Material

Supplementary Data
