# Supplementary material for: Pipit: visualizing functional impacts of structural variations
Source: Bioinformatics. 2013 Jun 25;29(17):2206–7. doi: 10.1093/bioinformatics/btt367 (PMC3740631; doi:10.1093/bioinformatics/btt367)
Supplement: Supplementary Data [file supp_29_17_2206__index.html]

Pipit: visualising functional impacts of structural variations — Pipit: visualizing functional impacts of structural variations — Pipit: visualizing functional impacts of structural variations — Supplementary Data 

# Pipit: visualizing functional impacts of structural variations

## 

files

**Files in this Data Supplement:**

- Supplementary Data - pdf file
