# Supplementary material for: Pipit: visualizing functional impacts of structural variations
Source: Bioinformatics. 2013 Jun 25;29(17):2206–7. doi: 10.1093/bioinformatics/btt367 (PMC3740631; doi:10.1093/bioinformatics/btt367)
Supplement: Supplementary Data [file supp_btt367_supplementaryMaterial.pdf]

BIOINFORMATICS

Vol. 00 no. 00 2012
Pages 1

Pipit: visualising functional impacts of structural variations

Ryo Sakai [1,2], Matthieu Moisse [3,4], Joke Reumers [5] and Jan Aerts [1,2]

[1]KU Leuven, Department of Electrical Engineering-ESAT, SCD-SISTA, Leuven, Belgium
[2]iMinds Future Health Department, Belgium
[3]Vesalius Research Center, VIB, Leuven, Belgium
[4]Laboratory of Translational Genetics, Department of Oncology, KU Leuven, Leuven, Belgium
[5]Janssen Infectious Diseases - Diagnostics, Janssen Pharmaceuticals, Beerse, Belgium

Received on XXXXX; revised on XXXXX; accepted on XXXXX

Associate Editor: XXXXXXX

1 SUPPLEMENTARY MATERIAL

1.1 Visual Encoding

Each affected gene is represented as a disk and filled according to which part of its structure is influenced by a structural variation(SV) (Fig. 1). If the left end of a gene structure is affected by a SV event, the left side of disk is coloured (Fig. 1A). If the right end of a gene structure is affected, the right side of disk is coloured (Fig. 1B). If a SV event affects the middle of gene structure including at least one exonic region, a vertical line in the middle of disk is drawn (Fig. 1C). If a SV event covers the entire gene structure, the disk is filled (Fig. 1D). If a gene structure is affected by multiple events, the affected regions are represented by overlaying on top of each other, and a black dot in the middle is drawn(Fig. 1E). In case of deletion, a potential fusion gene is presented as two circles with both inner halves coloured, as shown in the Fig. 1F.

1.2 CSV files

The user can load a comma-separated values (CSV) file with a header to compare against affected genes. The CSV file includes the Ensembl Gene ID and its categorical information. Two sample CSV files are available at https://bitbucket.org/biovizleuven/pipit. The first sample CSV file contains the haploinsufficiency sores for the human (Huang *et al.*, 2010). The second sample is a list of oncogenes for the mouse. The CSV file is specified when loading the data. The user can use the categorical information to search and highlight among affected genes. Example use cases are described below.

In Figure 2, structural variations of the human genome are visualised in Pipit and the CSV file of haploinsufficiency scores is loaded. Those affected genes that score higher than 0.8 are highlighted as shown on the right panel (Fig. 2B), and one of gene groups is selected as pointed out in Fig. 2A. In this *collapsed, ordered gene view*, the *TAF10* gene scores 0.8 in haploinsufficiency and its entire region is deleted. In Figure 3, Pipit visualises the same data as the above, but the layout is changed to the *unit plot view*. It sorts affected genes based on the type structural variations.

In Figure 4, structural variations of the mouse genome is visualised in Pipit and the CSV file of oncogenes are loaded. The categories of matched oncogenes are shown on the right panel and the *oncogenes* are selected(Fig. 4B). There is one affected oncogene and it is selected as pointed out in Fig. 4A, which is the *Met* gene.

1.3 Layouts

There are four layouts to explore the structural variation data. The default view is the *collapsed, ordered gene view*(Fig. 4). In the *expanded view* (Fig. 5), all affected genes are individually visualised. The *chromosome position view* (Fig. 6) shows affected variants mapped to their genomic positions. Lastly, the *unit plot view* (Fig. 7) visualises affected genes by their type of structural variant event, such as deletion, tandem-duplication and so forth. Each disk is animated in transition from one layout to another.

REFERENCES

Huang, N., Lee, I., Marcotte, E. M., and Hurles, M. E. (2010). Characterising and predicting haploinsufficiency in the human genome. *PLoS genetics*, **6**(10), e1001154.

© Oxford University Press 2012.

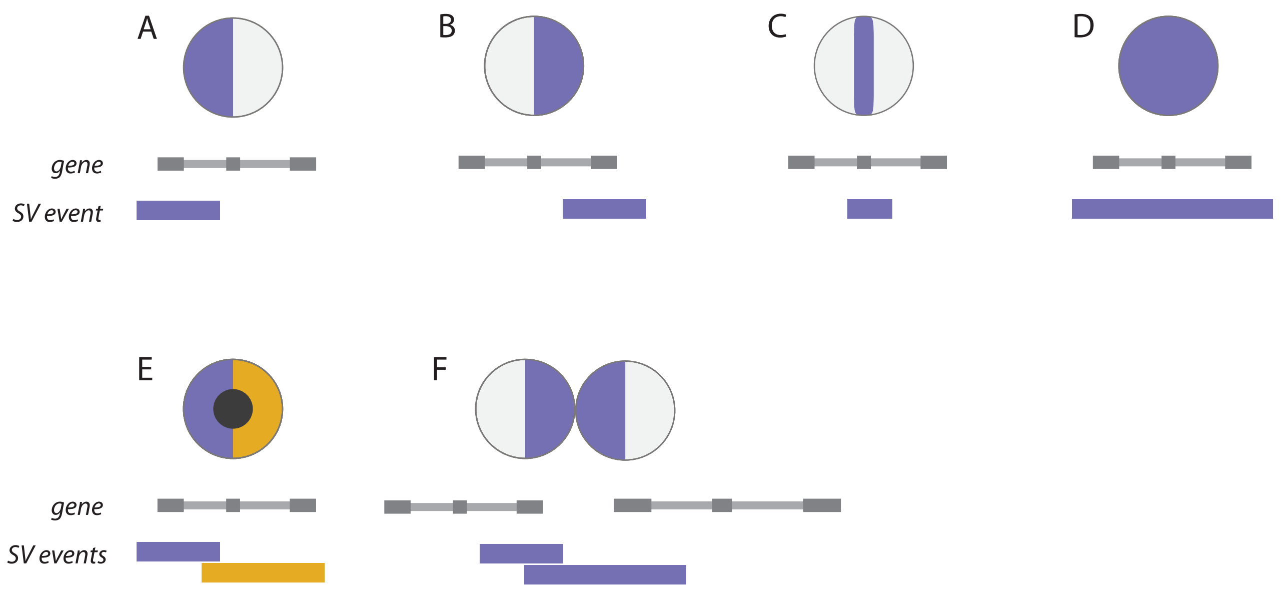

Fig. 1. Schematic illustration for the visual encoding. (A) The left end of gene structure is affected. (B) The right end of gene structure is affected. (C) The middle of gene structure, including at least one exome, is affected. (D) The entire gene structure is affected. (E) If the gene is affected by multiple structural variation events, each representation is overlaid and a dot in the centre is added. (F)If the blue corresponds to deletion events, this representation is an example of a potential fusion gene.

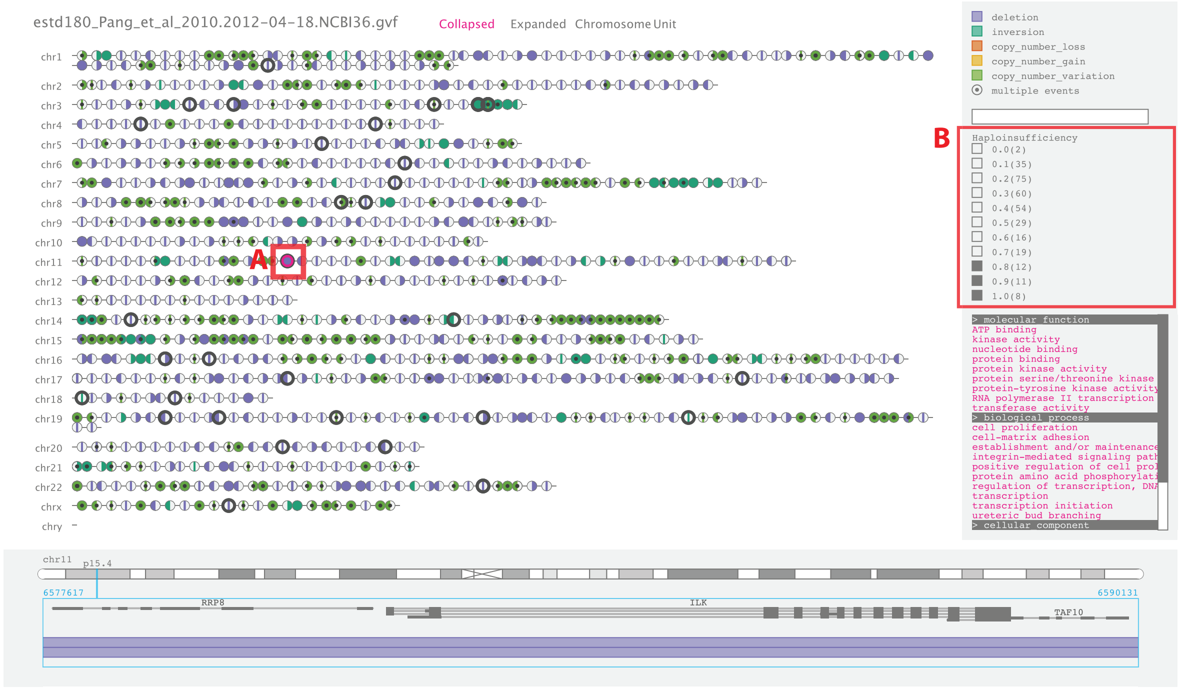

Fig. 2. Pipit visualising the structural variation data of the human genome, *estd180_Pang_et_al_2010.20120418.NCBI36.gvf*. (A) A group of genes is selected. Among the selected genes, the *TAF10* gene has the haploinsufficiency score of 0.8. (B) The haploinsufficiency score categories are shown and the scores higher than 0.8 are selected.

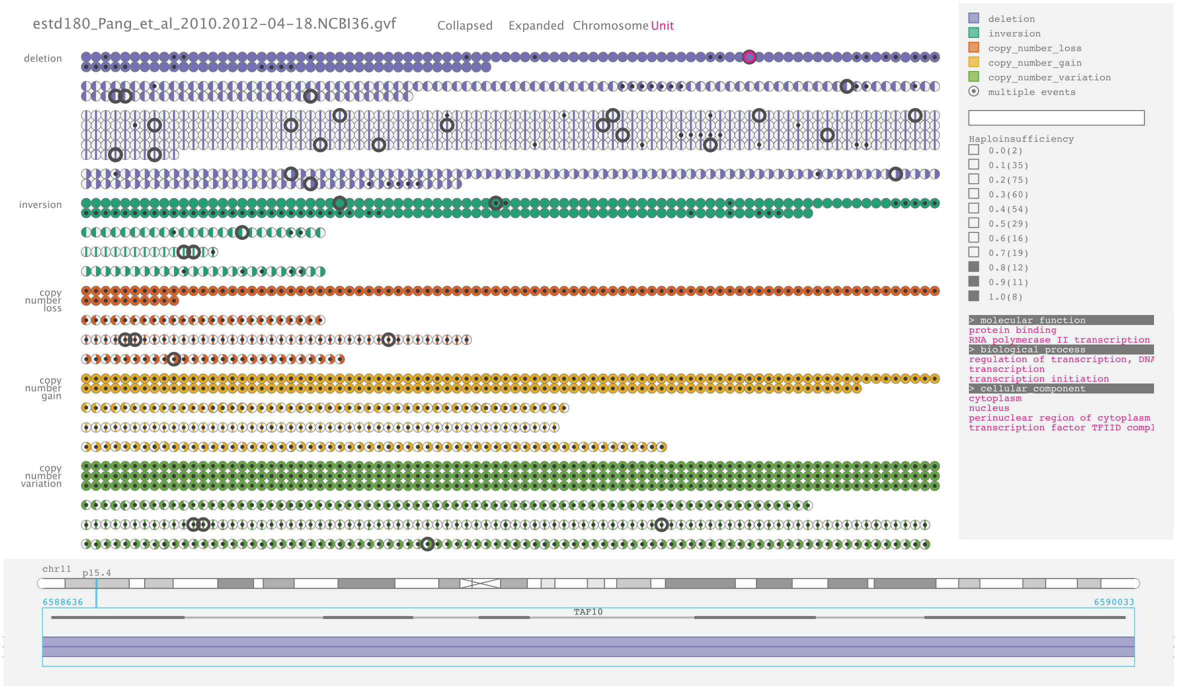

Fig. 3. Pipit visualising the structural variation data of the human genome, *estd180_Pang_et_al_2010.20120418.NCBI36.gvf* in the *unit plot view* layout.

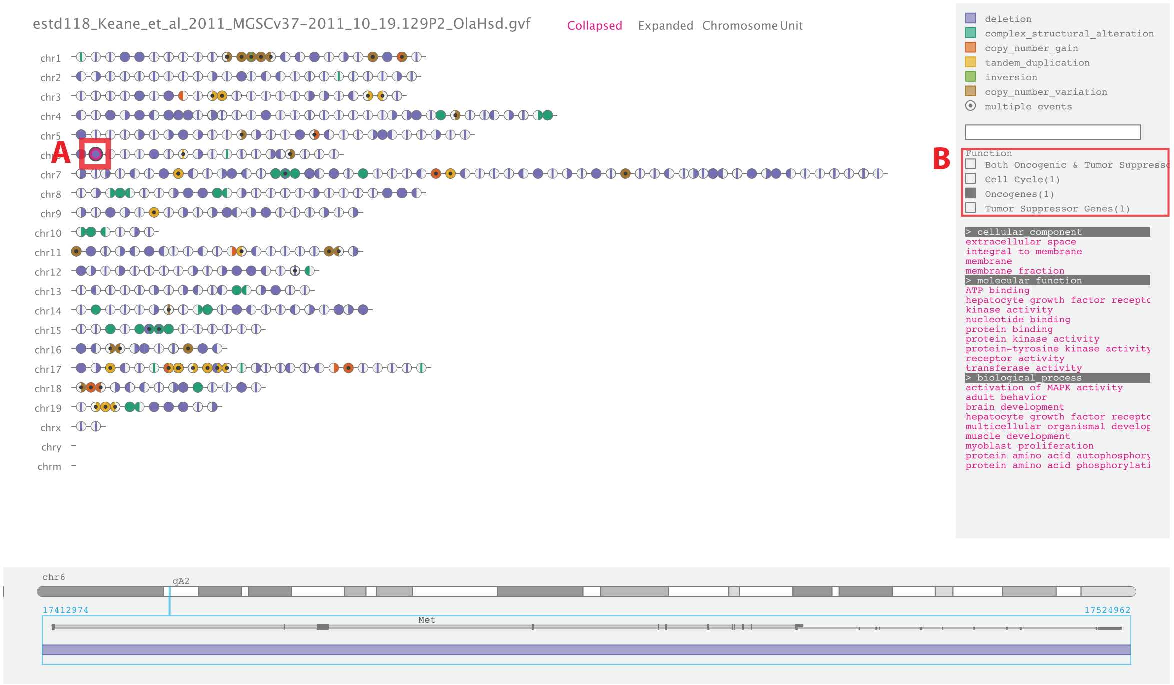

Fig. 4. Pipit visualising the structural variation data of the mouse genome, *estd118_Keane_e_a_2011_MGSCv372011_1_19İ29P2_OlaHsd.gvf*. (A)The *Met* gene is selected. (B) The categories of oncogenes, defined in the loaded CSV file, are listed. The *Oncogenes* category is selected.

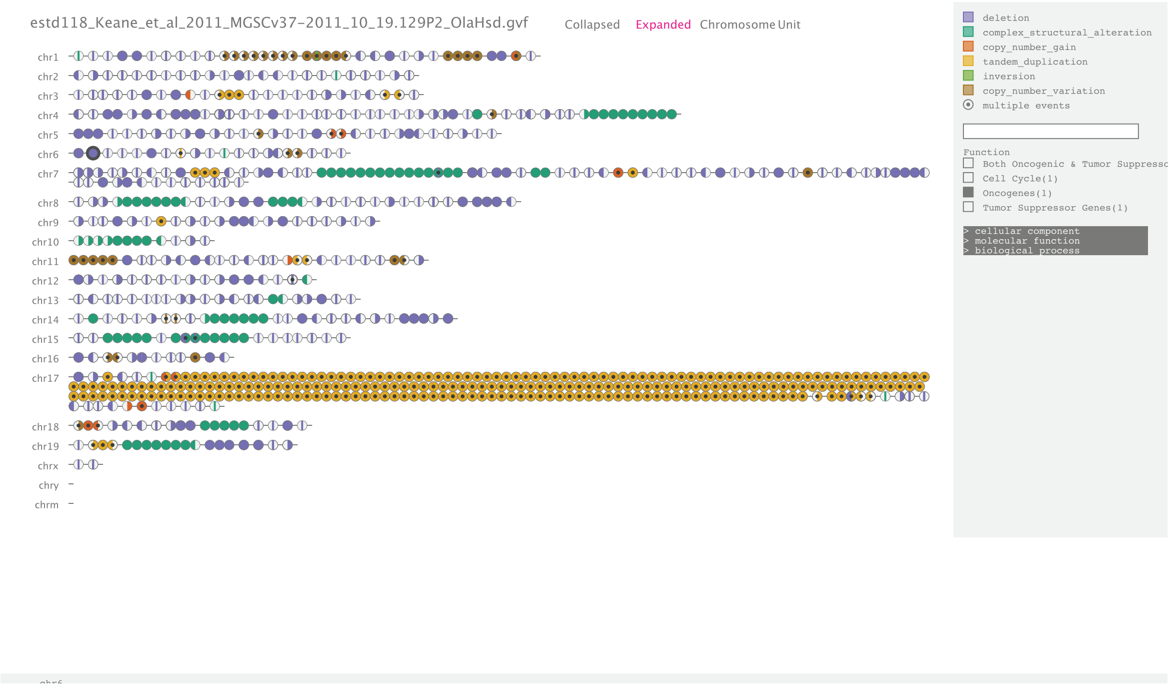

Fig. 5. Pipit visualising the structural variation data of the mouse genome, *estd118_Keane_et_al_2011_MGSCv372011_10_19.129P2_OlaHsd.gvf* in the *expanded view* layout. Each disk represents a gene. It shows a large number of genes on the chromosome 17 are tandem duplicated.

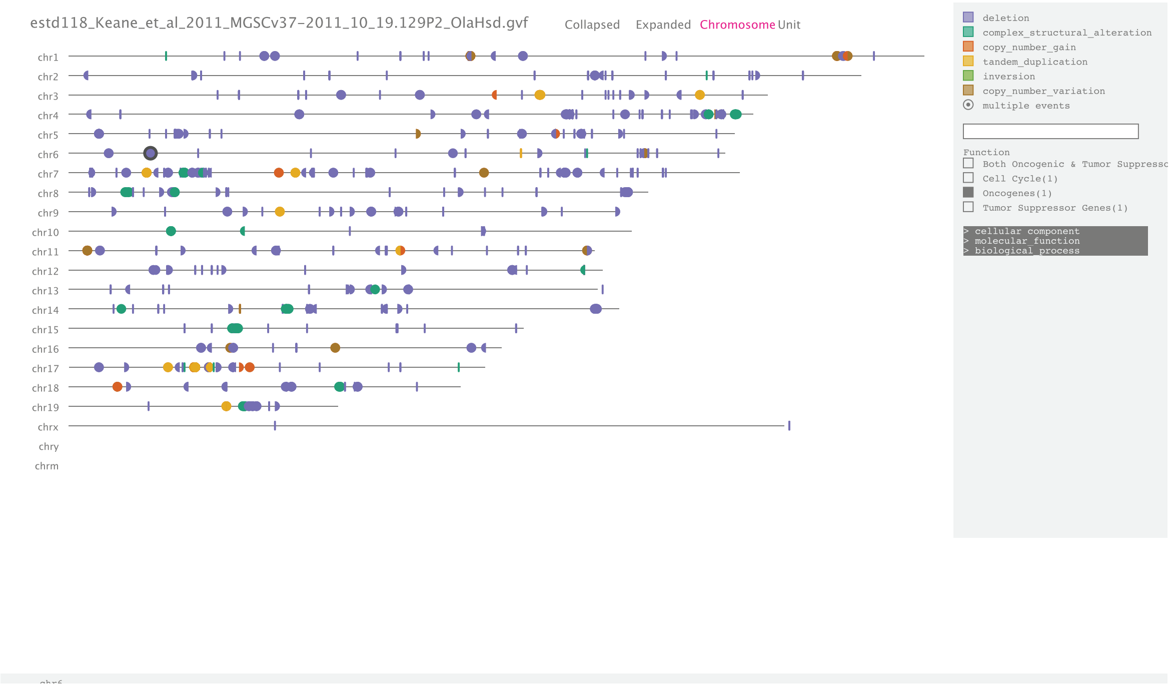

Fig. 6. Pipit visualising the structural variation data of the mouse genome *estd118_Keane_et_al_2011_MGSCv372011_10_19.129P2_OlaHsd.gvf* in the *chromosome position view*. The gene motifs are positioned based on their chromosomal position. The disk encoding is simplified by not drawing the outlining circle.

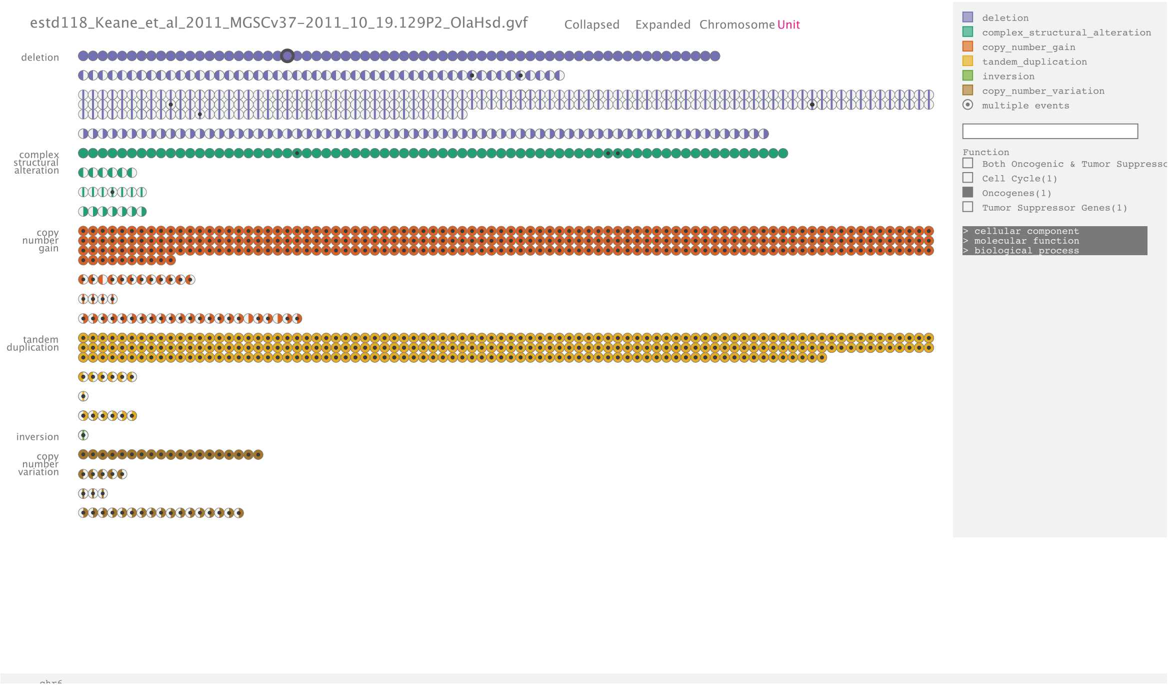

Fig. 7. Pipit visualising the structural variation data of the mouse genome, *estd118_Keane_et_al_2011_MGSCv372011_10_19.129P2_OlaHsd.gvf* in the *unit plot view* layout. Each affected gene is sorted based on the structural variation type and the affected regions.
